# Dreamers’ evaluation of the emotional valence of their day-to-day dreams is indicative of some mood regulation function

**DOI:** 10.3389/fnbeh.2022.947396

**Published:** 2022-09-16

**Authors:** Kheana Barbeau, Chloé Turpin, Alexandre Lafrenière, Emma Campbell, Joseph De Koninck

**Affiliations:** ^1^School of Psychology, University of Ottawa, Ottawa, ON, Canada; ^2^Department of Psychology, Université de Montréal, Montreal, QC, Canada

**Keywords:** function of dreams, dream mood regulation, desensitization in dreams, dream emotions, rating of dream emotional valence

## Abstract

Dreams may contribute to psychological adaptation by aiding in mood regulation. One way it could be achieved is through a desensitization process whereby negative events are replayed within the dream under lower conditions of negative emotionality. Evidence of this theory is supported by the tendency of dreamers to evaluate their emotions felt in their dreams more positively compared to an independent judge (i.e., positivity bias). Additionally, it has been observed that while dream emotions are typically more negative than pre-sleep emotions, morning emotions are more positive, suggesting that emotional regulation occurs overnight and may help improve mood in the morning. The present study aimed to examine the relationships between pre-sleep, dream, and morning mood and the potential desensitization function of remembered dreams as indicated by their effects on morning mood and stress.

**Methodology:** Participants (*N* = 188; Mean age = 19.2, *SD* = 3.0) recorded their dreams (*N* = 345 dreams) and self-reported their stress and mood at bedtime, during their dream retrospectively, and upon waking. A judge also evaluated the subjects’ dream moods. Subjects’ positivity bias was defined as the difference between the subjects and the judge’s evaluation of the positive emotions in the dream.

**Results:** A MANOVA revealed that subjects perceived a higher level of positive emotions in their dreams compared to a judge. Multi-group path analysis revealed that some relationships between pre-sleep, dream, and morning emotions and stress differed in positive and negative dream nights. In both groups, the strongest predictors of morning mood and stress were pre-sleep mood and stress, respectively. The second strongest predictor of positive morning mood was the subjects’ dream positivity bias.

**Conclusion:** Results provide some support for the association of dreaming in mood regulation attributable to REM sleep. They also highlight that pathways implicated in mood regulation may be distinct from stress regulation.

## Introduction

The extensive research on dream formation has shown empirical support for the presence of some continuity between waking life and dream content. It has been postulated that dreams tend to reflect waking life experiences. It has also been suggested that the incorporation of waking-life events in dreams could have implications on an individual’s psychological adaptation to these experiences. These notions have become key postulates of the continuity hypothesis of dreams (CH), which is now an umbrella theory first described in detail by Hall and Nordby (1972). Regarding the CH, the term continuity is very general and has thus encompassed several hypotheses and, at times, conflicting interpretations regarding the formation and function of dreams. Some researchers have postulated that the continuity was in the dreamer’s cognitive activity, such as their perceptions and concerns (Domhoff, 2018). Schredl and Hofmann (2003) argued that waking-life experiences and events influenced dream content more generally, suggesting that dreamers’ specific concerns and preoccupations were not uniquely represented in dreams. Modern research tends to show that all of these elements (i.e., thoughts, perceptions, preoccupations) and daily activities can be found in dream content since these concepts are often difficult to dissociate (Schredl, 2019). Research has shown that various aspects of waking life are incorporated into the content of dreams. Examples include social roles (Lortie-Lussier et al., 1985), gender (Dale et al., 2016), physical health (King and DeCicco, 2007), mental health (Schredl and Montasser, 1999) personality and psychotherapy process (Koulack et al., 1976; Busby and De Koninck, 1980; Hartmann et al., 1991; Samson-Daoust et al., 2019), remote past experiences (Grenier et al., 2005) and cognitive capacity (Fogel et al., 2018). Other studies have observed that certain chronobiological determinants, such as hormonal fluctuations (Wiebe et al., 2007), and certain major life changes, such as pregnancy (Sabourin et al., 2018), can influence dream content and result in dream modulations (e.g., affective changes, element incorporations). One important common denominator is that dream incorporations of waking life are typically distorted, such that independent observers are unable to detect a clear resemblance between participants’ daily events descriptions and manifest dream content reports (Roussy et al., 2000). This is unsurprising since some types of day activities, typically cognitive activities, such as reading, writing, and counting, are seldomly reported in dreams (Hartmann, 1998) and there is essentially no episodic memory in the manifest content of dreams. Finally, new experiences, such as learning a second language, can take several days or weeks to be incorporated into dreams; therefore, there is also a temporal aspect to consider when examining the degree of continuity between waking life and dreams (De Koninck et al., 1990).

De Koninck (2012) has proposed a hierarchy, in terms of importance, of the contributions of the different factors and components that shape the construction of dreams. Consistent with this layered approach is the increasing evidence that, once the proper physiological substrate and cognitive capacity are in place, dream construction prioritizes emotional waking-life experiences and concerns with a negative bias (Malinowski and Horton, 2014; Domhoff, 2019). It is thus not surprising that traumatic events and major life changes have also been found to have a significant impact on dream content. Events, such as divorce (Cartwright, 1991), sexual and physical abuse (Belicki and Cuddy, 1991), and torture and war (Lavie and Kaminer, 1991; Valli et al., 2006) have been shown to have a significant impact on emotions in the content dreams of individuals who have experienced these events. Results from more recent studies examining the impacts of COVID-19 on dreams demonstrate that individuals who were more strongly affected by the pandemic physically, socially, or psychologically had more negatively toned dreams (Schredl and Bulkeley, 2020). Finally, anticipatory stressful events, for example, an academic examination, et al., has been found to be incorporated into a recent dream, such that many students dreamt of forgetting answers or being late for the exam the night before (Arnulf et al., 2014). Furthermore, students who dreamt of the exam the night before performed better on the exam (Arnulf et al., 2014), demonstrating that episodic simulation during dreaming has an adaptive value and that there is a degree of continuity between anticipatory feelings during waking and in dreams as suggested by Lemyre et al. (2022).

While it is thus well documented that waking life can shape dream formation and that the most significant relation resides in the emotional dimension with its accompanying mood, a more challenging dimension of the CH is whether there is a relationship between dreams and subsequent waking life. This notion has led to dream function theories in relation to the impact of dreams on waking life (Kuiken and Sikora, 1993) and their potential adaptive role. It is well established that nightmares have a negative impact on waking life (Nixon et al., 2017); however, less is known on how dreams could contribute to psychological adaptation in average waking life, such as daily mood or stress. Studies starting in the 1960s and 1970s have explored the dreams of individuals who had been exposed to naturally or experimentally induced stress (Breger et al., 1971; Cohen and Cox, 1975; De Koninck and Koulack, 1975) in order to test dream function theories that ranged from mastery to compensation (Dallett, 1973). For example, Breger (1967) proposed that dreams “serve to integrate affectively aroused material into structures within the memory system that have previously proved satisfactory in dealing with similar material and serve a unique function in the assimilation and mastery of arousal material into the “solutions” embodied in existing memory systems.” Some studies supported the mastery hypothesis (Cohen and Cox, 1975), while others did not (De Koninck and Koulack, 1975; Koulack et al., 1985). The alternative hypothesis was that dreams serve an adaptive function through a compensating mechanism of needs arising from the presleep experience (PE). For example, Foulkes et al. (1967) reported that following the presentation of a baseball film, the dreams of young males were more aggressive than the dreams following a Western film, suggesting compensation for the hostility dimension. Wright and Koulack (1987) proposed a disruption-avoidance model combining these approaches. Despite their divergence on the interpretation of dream function, these two approaches share the postulate that dreams can act as a “safe space” and help individuals to explore and resolve emotional problems (Hartmann, 1998). Aligned with this notion, Cartwright (2001) reported that newly divorced individuals who dreamt about their spouse better adapted to their new situation. Other researchers have explored the potential mood regulation function of dreaming given that emotions play an important role in dream formation.

While in the early 70s most theories were focused on dream content itself as an agent of adaptation, studies conducted by Kramer and colleagues demonstrated that successive REM dreams during the night progressively improved mood and thus served an adaptive role (Kramer and Roth, 1972; Kramer et al., 1974). This is achieved through the content of the dream. These observations lead to his mood regulation theory elaborated in future works (Kramer, 1993, 2007). He proposed that “the physiological and psychological activities during sleep appear to be corrective like a thermostat operating to move the mood level toward a central and lower point” (Kramer, 2007, p. 183). Perlis and Nielsen (1993) more specifically suggested that a process of desensitization was taking place during REM dreams, notably with the reduction of muscle tone. In agreement with Kramer (1993), Kramer (2007); they generalized the function of REM sleep to the function of dreaming. It also did not limit the adaption process to rely on the specific type of content, such as mastery or compensation, but on a more encompassing process. Therefore, it is proposed plausible that dreams may be responsible for sensory and affective integration and the process of desensitization as indicated by the increased subjective perception in the pleasantness of emotionally intense dreams would be enabled by specific processes that occur during REM sleep such as muscle relaxation and or positive emotions.

This notion is consistent with the consensus that REM sleep plays a pivotal role in the processing of emotional waking-life experiences by contributing to emotional memory consolidation (Breger, 1967). Not surprisingly, Kramer (2007) suggests uninterrupted REM sleep is more conducive to better morning mood regulation than when there are awakenings from REM sleep with dream recall (for example, Koulack et al., 1985). To some extent, neuroimaging studies have found some overlap in the neural substrates that regulate dreaming and their emotional salience and those involved in emotion regulation during waking (Scarpelli et al., 2019). EEG studies also found that theta activity patterns during REM sleep in individuals who had incorporations of memories in their dream experience were similar to patterns observed when memory processing is occurring during wakefulness, suggestive of a continuation of emotional processing of waking-life events in sleep cycles where dreaming is most likely to occur (De Gennaro et al., 2012; Scarpelli et al., 2015). Levin and Nielsen (2009) refined this model, which is currently known as the Neurocognitive Model of Nightmares (NMN) and propose that dreams regulate fear-infused emotions by a recombination of fearful memories with non-fearful mnesic elements. More recently, a study by Malinowski and Horton (2014) further explored the potential emotion regulation function of dreams. They found that elements of waking life that were consistently incorporated in dreams were significantly more emotional, but not necessarily more stressful. Malinowski and Horton (2014) also suggested that the preferential incorporation of emotional experiences into dreams may contribute to psychological adaptation in various ways. For instance, it could aid in problem-solving and help individuals derive a sense of mastery over affectively arousing dreams. Regarding emotion regulation, in particular, this incorporation process could help ameliorate emotions attached to arousing waking-life experiences, which, in turn, may reduce arousal to this event in waking-life. The benefits of emotional processing occurring during dreaming have been demonstrated in those who suppressed their unpleasant thoughts: they were more likely to experience dream rebound compared to those who suppressed pleasant thoughts, which in turn, had a therapeutic effect by providing a more pleasant perspective on unpleasant thoughts (Malinowski et al., 2019). Furthermore, Vallat et al. (2018) also observed that dreams contribute to emotion regulation, such that subjects whose dreams reflected their waking-life experiences perceived their dreams more positively than the actual event. This finding is further corroborated by our recent study that demonstrated that subjects who had experienced a recent troubling life event had a higher level of negative emotions but similar levels of positive emotions in their dreams compared to those who did not have a recent troubling experience (Barbeau et al., 2022). Both of these studies support the notion that dreams can contribute to psychological adaption by aiding in the integration and consolidation of emotions through a process of desensitization, which may have the potential to positively affect subsequent waking-life. However, it remains unknown how this positive dream affectivity present even during negatively toned dreams influences subsequent waking states, such as morning mood and stress. An interesting observation that may be indicative of the desensitization process during dreaming are studies reporting that dreamers tend to self-evaluate their emotions in dreams more positively than independent judges who read their dream narrative (Schredl and Doll, 1998; Sikka et al., 2014, 2017, Sikka et al., 2018, 2021). Studies assessing this phenomenon have found that the discrepancy can be affected by the personality of the dreamer, the length of the dream report, and potentially the instructions given to the external raters (Röver and Schredl, 2017). For instance, Röver and Schredl (2017) observed that judges underestimate the intensity of dream emotions, specifically positive ones, and the word length of the dream report was inversely associated with the discrepancy between the subjects’ and evaluators’ ratings of negative dream emotions only. This finding was interpreted as a potential artifact elicited by either methodological design (i.e., dream recall instructions not specifying for subjects to focus on reporting their emotions felt while dreaming; Sikka et al., 2017) or subjects’ tendency to underreport positive emotions in their dream reports, particularly when their reports are shorter in length (Röver and Schredl, 2017). Nonetheless, these observations have been interpreted by others as an illustration of a positive bias within the dream experience, which recently has been found in waking mind wandering (Sikka et al., 2021). Essentially, the literal objective content is in contrast with the subjective emotional experience of dreams, which would contribute to its adaptive value. In a similar fashion outlined by Scarpelli et al. (2019), Sikka et al. (2021) proposed a neural basis of affective experience and the role of the default-mode network (DMN) and the prefrontal cortex (PFC) in the management of subjective experiences. Finally, what gives credence to the notion of subjects’ positive bias is the very well-documented presence of negative components in dreams beyond emotions. Indeed, the first normative study of dream content by Hall and Van De Castle (1966), which has been replicated in a normative Canadian population (Dale et al., 2016), confirms the predominance of aggression over friendliness, failures over success and misfortune over fortune in dreams. However, the interpretation of these events may differ depending on the evaluator [i.e., self (subject) vs. external evaluator] and thus the evaluation of emotions felt during these dreams may also consequently differ. Of interest to the present study, the extent of this positive bias could then serve as a tool to assess the impact that desensitized dream experiences have on morning emotional states, such as improved mood (e.g., more positive morning emotions) and/or lowered stress.

### Hypothesis and predictions

The objective of the present study was to continue to assess the relationships between the dreamer’s mood and stress prior to sleep and the emotional experience during the dream, while also examining the relationship between these pre-sleep and remembered dream states on waking mood and stress.

When testing dream function theories, it is essential to evaluate the post-sleep experience and go beyond extrapolating from dream content in relation to the preceding waking experience. Early studies seeking to examine the relationship between dreaming and subsequent morning waking states attempted to experimentally manipulate dreams by exposing subjects to external stimulation during REM sleep episodes (De Koninck and Koulack, 1975) or exposing them to pre-sleep stimulations followed by REM dream report collections (Cohen and Cox, 1975). These studies have yielded inconclusive results due to eliciting sleep disruptions and the effect of these disruptions on dream recall and morning mood. For example, Koulack et al. (1985) observed that morning mood was significantly more positive following uninterrupted sleep compared to a REM dream collected during the night regardless of dream valence. For the present study, it was decided to use a correlational model applied used in previous studies (Schredl and Reinhard, 2010; Nixon et al., 2017) to elaborate on the relationship between pre-sleep, dream, and morning emotions. This allowed us to determine which is the best predictor of subsequent morning mood and stress using a protocol of normal dream diaries without manipulation and without taking into account dream content. We also attempted to examine the notion of the adaptive function of dreaming on emotion and stress regulation through desensitization as assessed by dreamers’ positive bias of their recalled dreams. In a stepwise approach, we attempted to replicate previous findings that found a discrepancy between dreamers and independent judge’s ratings of positive emotions in subjects’ recalled dreams (i.e., positivity bias) while taking into consideration artifacts identified in previous studies, such as dream length and mood rating methods, notably by establishing the reliability of external judges. Thus, we assessed whether this bias is a useful indicator of the desensitizing power of the dream as indicated by its association with positive morning emotions and lower morning negative emotions and stress.

According to mood regulation theories of dreams we predicted that dreamers would evaluate the content of their dreams more positively than independent judges. More specifically, there should be a significant discrepancy between the emotions present in the dreamer’s dream narrative as assessed by independent judge and the emotions that the dreamer reported feeling.

According to the CH, there is a certain degree of continuity between waking-life states and dream states; therefore, we hypothesized that pre-sleep emotions and stress would be associated with dream emotions. We further extended the postulates of the CH to morning states and hypothesized that pre-sleep emotions and stress and dream emotions would be associated with morning emotions and stress.

Dreaming should facilitate the desensitization to negative waking-life events through simulations in recalled dreams, which aids psychological adaptation in waking-life. Therefore, we predicted that there would be stronger positive associations between positive dream emotions and positive morning emotions compared to positive pre-sleep emotions and positive morning emotions. Furthermore, the process of desensitization and its adaptive value were gauged by examining the predictive power of dreamers’ positivity bias on morning mood and stress. We predicted that dreamers’ positivity bias would be the strongest predictor of positive morning emotions and would contribute to lower morning stress as indicated by a negative association. Considering that much of the research examining the emotion regulation function of dreams has been conducted in individuals who experienced traumatic or adverse events, who in turn typically have more negative dreams compared to normative populations, we predicted that the desensitization process, an indicated through dreamer’s positivity bias, may have different impacts on dreamers waking mood and stress depending on whether they have a positive or negative dream night.

## Method

### Participants

One hundred eighty-eight participants were selected from a pool of participants that was previously collected in a larger study examining normative dreams among Canadians between 2004 and 2017 (Dale et al., 2016), which was before the COVID-19 pandemic. Male (*n* = 90; 48%) and female participants (52%) were between the ages of 12–24 years old (Mean = 19.2, *SD* = 3.0). Most reported on two dreams (*n* = 157; 84%). Participants were recruited through advertisements (e.g., at a university and on social media), word of mouth (e.g., personal contacts at school boards, at public presentations and conferences), and through retiree associations. Participants were unaware of the purposes of the study and provided written consent. The study was approved by the Research Ethics Board (REB) at the University of Ottawa.

After obtaining participants’ consent, they were instructed to complete a dream questionnaire using pen and paper at home until they reported at least one dream for a maximum period of 10 days. The dream questionnaire (DQ), which was developed for the Normative Study on the dreams of Canadians (Dale et al., 2015, 2016, Dale et al., 2017), comprised several sections. Of particular interest to this study, were the data from the sections related to participants’ emotions and stress experienced in the evening before sleeping and upon waking in the morning, and sections related to aspects of their (recalled) dreams, such as the narrative of their dream and the emotions in their dream. Further descriptions of these subsections of the DQ used in the current study are described below.

### Measures

#### Sociodemographic questionnaire

The DQ included a sociodemographic questionnaire. Participants self-reported their age, gender, marital status, profession, and education.

#### Pre-sleep stress and morning stress

A section of the DQ measuring aspects of waking life included questions related to levels of stress experienced in the evening at bedtime. On a Likert scale ranging from 0 (*none*) to 4 (*very high*), participants rated their current level of stress. Participants also reported their level of stress upon waking using the same scale. In the current study, participants’ scores on pre-sleep stress and morning stress represent their level of stress at bedtime and upon waking on nights when they had dreams.

#### Pre-sleep emotions and morning emotions

The section of the DQ measuring aspects of waking life and stress before bedtime also contained a mood checklist developed by Folkman and Lazarus (1985) measuring the subject’s current levels of 15 positive and negative emotions. On a Likert scale ranging from 0 (*not at all*) to 3 (*a lot*), subjects rated the degree to which the following emotions were experienced before bedtime: worried, fearful, anxious, angry, sad, guilty, disappointed, disgusted, exhilarated, happy, pleased, relieved, confident, hopeful, and eager. Subjects completed the mood checklist again upon waking to measure participants’ positive and negative morning emotions. Subject’s positive and negative pre-sleep and morning emotions scores were transformed from a 0 to 3 to a 1 to 4 scale (i.e., a 0 became a 1, a 1 became a 2, a 2 became a 3, and a 3 became a 4) to be on the same scale as our other questionnaires, such as our measures of stress and dream emotions. After re-scaling, the subject’s pre-sleep positive emotions score was created by averaging their ratings on exhilarated, happy, pleased, relieved, confident, hopeful, and eager before bedtime. Subject’s pre-sleep negative emotions score was created by averaging their ratings on worried, fearful, anxious, angry, sad, guilty, disappointed, and disgusted before bedtime. Similarly, the subject’s morning negative emotions score was created by averaging their ratings on worried, fearful, anxious, angry, sad, guilty, disappointed, and disgusted upon waking. Subject’s morning positive emotions score was created by averaging their ratings on exhilarated, happy, pleased, relieved, confident, hopeful, and eager upon waking. Cronbach’s alpha for the pre-sleep negative emotions and positive emotions scores were 0.79 and 0.82, respectively. Cronbach’s alpha for the morning negative emotions and positive emotions scores were 0.83 and 0.81, respectively.

#### Dream reports and dream emotions

Following the section of the DQ related to waking life experiences and pre-sleep emotions and stress, participants completed the morning section of the DQ. This section comprised the description of the narrative of their dream immediately upon waking followed by assessing their emotions experienced in their dream. Using a four-point Likert scale (1 = *not at all*, 2 = *a little*, 3 = *moderate*, 4 = *a lot*) was used to assess the five emotions found in the scale developed by Hall and Van De Castle (1966) with the added anxiety dimension. More specifically, participants rated the degree to which they experienced joy, happiness, apprehension, anger, sadness, fear, and anxiety in their dream. Subjects’ evaluations of the positive emotions in their dream were created by averaging their ratings on joy and happiness. Subjects’ evaluations of the negative emotions in their dream were achieved by averaging their ratings on apprehension, anger, sadness, fear, and anxiety. Cronbach’s alpha was 0.94 for the subject’s mean score of positive dream emotions and 0.62 for the subject’s mean score of negative dream emotions.

Considering that we were interested in examining the potential discrepancy between a subject’s and a judge’s rating of oneiric emotions, an independent judge also evaluated the degree of positive and negative emotions in subjects’ dream narratives. After being trained in scoring emotions in dream narratives, the independent judge scored the degree of positive and negative emotions present in subjects’ dream narratives using the same Likert scale that was used by the subjects. The independent judge was one of several whose reliability of scoring was assessed against another judge. Due to the high level of agreement between the two judge’s ratings (i.e., each positive and negative dream emotion level rating), only one judge’s evaluations were used in the current study. The independent judge was blind to the subjects’ evaluations of positive or negative emotions experienced in their dream to ensure that their evaluation remained unbiased. The judge’s evaluation of positive emotions in the dream was created by averaging their ratings on joy and happiness. The judge’s evaluation of negative emotions in the dream was created by averaging their ratings on apprehension, anger, sadness, fear, and anxiety. Cronbach’s alpha was 0.95 for the judge’s mean score of positive dream emotions and 0.70 for the judge’s mean score of negative dream emotions.

To assess whether subjects display a positivity bias when recalling their dream emotions, a score was created to represent the discrepancy between the subject’s and judge’s ratings of the level of positive emotions in the dream. This score was computed by subtracting the subject’s mean positive dream emotions score from the judge’s mean positive dream emotions score. If this computation resulted in a negative score, denoting that the subject’s mean positive dream score was lower than the judge’s mean positive dream score, the subjects’ positivity bias score was then transformed to a 0 to represent the absence of a positivity bias (*n* = 132 dreams originally had no presence of a positivity bias; *n* = 53 additional scores were transformed to a 0 due to a negative score).

Since we conducted our analyses on positive and negative dreams separately, we decided to use an objective categorization of emotional valence. Thus, we used the difference between the judge’s positive and negative emotions scores to dichotomize the dreams according to their global emotional valence. A negative score suggested that the subject had a more negative dream, a positive score suggested that the subject had a more positive dream, and a score of 0 suggested that the subjects had a dream with equal levels of positive and negative emotions.

### Data analytical plan

To examine whether subjects possess a positivity bias when perceiving their emotions in their dreams, a between subjects MANOVA was conducted to examine if there were differences in the subjects’ and judge’s ratings of the positive and negative emotions in the dreams. An *a priori* power analysis using G^*^Power (Faul et al., 2007) recommended a sample size of 158 participants for detecting a medium effect size with power of 0.80 and an alpha of 0.05. To reduce Type 1 error from conducting multiple comparisons, a Bonferroni adjustment was applied to each *post hoc* comparison (0.05/2 = 0.025). Due to previous studies finding an association between word length of the dream report and magnitude of the discrepancy between a subject’s and judge’s evaluations of positive dream emotions, we randomly selected 50 subjects who had a more negative dream and 50 subjects who had a more positive dream and assessed whether word length was associated with subjects’ positivity bias score by conducting a Pearson’s Correlation. To examine the associations between pre-sleep stress and emotions, dream emotions, including subjects’ positivity bias, and morning stress and emotions, a path analysis using Mplus Version 7 was conducted. For this analysis, we used the judge’s ratings of the positive and negative emotions in the dreams because they were considered more objective, while also examining the contribution of the subject’s positivity bias (i.e., a score representing the discrepancy between the subject and judge’s ratings of the positive emotions in the dream) on morning emotions. Furthermore, we sought to examine whether these associations were similar across nights where subjects had more positive or negative dreams. Thus, we tested the tenability of our path model and the invariance (equivalency) between the correlations observed in these paths (parameters) across these two groups using a multigroup approach in Mplus.

To test the invariance in parameters across positive and negative dream nights in the multigroup path analysis, a constrained model was tested against an unconstrained model. In the constrained model, parameters were constrained to be equivalent across groups (i.e., across dream nights), whereas in the unconstrained model, parameters were free to vary. The constrained and unconstrained models were then compared using a chi-square difference (χ^2^diff) test to determine whether global dream valence played a moderating role on the path model. A significant chi-square difference would support the notion that dream valence plays a moderating role on the path model and that the parameters are not equivalent across dream nights. In the event of this result, follow-up analyses testing each parameter separately for invariance by unconstraining the relationship between variables would be conducted; the χ^2^diff resulting from this would be compared to the constrained model whereby *p* < 0.05 suggests invariance across the groups (i.e., dream nights).

For all models generated in the study (e.g., constrained, unconstrained, final accepted model), model fit was considered good if the χ^2^ value was nonsignificant (Barbeau et al., 2019), the comparative fit index (CFI) and Tucker-Lewis index were ≥0.90 (Forza and Filippini, 1998; Hair et al., 2010), (b) the root mean square error of approximation (RMSEA) was ≤0.06 (Barbeau et al., 2019), and the standardized root mean square residual (SRMR) index was ≤0.08 (Hu and Bentler, 1999).

## Results

Univariate outliers were winsorized. The assumption of multivariate normality, which was assessed by examining scatterplots, equality of covariances MANOVA (Box’s M, *p* < 0.001), and multivariate outliers were violated. The multivariate outlier was retained because the presence and absence of the outlier had no effect on the MANOVA results. Furthermore, MANOVA is robust to departures of normality and equality of covariances; therefore, this analysis was still pursued. Regarding the assumptions of the multi-group path analysis, *n* = 11 participants were considered multivariate outliers according to their group membership (i.e., dream valence) and were removed from the analysis.

Overall (*N* = 334 dreams), *n* = 205 subjects’ dreams were considered more negative, *n* = 117 subject’s dreams were considered more positive, and *n* = 12 subject’s dreams were considered to be equally mixed based on the judge’s score of positive and negative dream emotions. Additionally, of those included in the path analysis model (*N* = 322), 137 subjects exhibited a positivity bias (*n* = 81 subjects who had a more negative dream and *n* = 56 subjects who had a more positive dream). For the multi-path analysis, the invariance model was tested between those who were considered to have more positive or more negative dream nights only. Means, standard deviations, and correlations among the variables in the model in the positive and negative dream night group appear in Tables s 1, 2, respectively.

**Table 1:** Means, standard deviations, and correlations among the variables in those who had positive dream nights.

Variable	Mean	SD	2	3	4	5	6	7	8	9
1. Pre-sleep positive emotions	2.3	(0.7)	−0.31*	−0.30*	−0.02	−0.10	−0.01	−0.37*	−0.07	−0.15
2. Pre-sleep negative emotions	1.5	(0.4)	-	−0.54*	−0.08	−0.14	−0.04	−0.09	−0.44*	−0.18
3. Pre-sleep stress	1.1	(1.0)	-	-	−0.10	−0.14	−0.01	−0.18	−0.20*	−0.32*
4. Positive dream emotions	2.6	(0.7)	-	-	-	−0.29*	−0.38*	−0.13	−0.10	−0.10
5. Negative dream emotions	1.4	(0.4)	-	-	-	-	−0.06	−0.27*	−0.15	−0.03
6. Subject’s positivity bias	0.5	(0.6)	-	-	-	-	-	−0.16	−0.02	−0.04
7. Morning positive emotions	2.1	(0.7)	-	-	-	-	-	-	−0.16	−0.03
8. Morning negative emotions	1.3	(0.4)	-	-	-	-	-	-	-	−0.31*
9. Morning stress	0.9	(0.9)	-	-	-	-	-	-	-	-

N = 117 dreams, *p < 0.05.

**Table 2:** Means, standard deviations, and correlations among the variables in those who had negative dream nights.

Variable	Mean	SD	2	3	4	5	6	7	8	9
1. Pre-sleep positive emotions	2.2	(0.7)	−0.34*	−0.27*	−0.02	−0.16*	−0.06	−0.54*	−0.14*	−0.02
2. Pre-sleep negative emotions	1.5	(0.5)	-	−0.68*	−0.05	−0.21*	−0.08	−0.24*	−0.56*	−0.42*
3. Pre-sleep stress	1.1	(1.0)	-	-	−0.04	−0.16*	−0.10	−0.17*	−0.47*	−0.44*
4. Positive dream emotions	1.1	(0.3)	-	-	-	−0.06	−0.09	−0.07	−0.04	−0.03
5. Negative dream emotions	2.3	(0.5)	-	-	-	-	−0.03	−0.14	−0.16*	−0.17*
6. Subject’s positivity bias	0.5	(0.8)	-	-	-	-	-	−0.28*	−0.11	−0.03
7. Morning positive emotions	1.8	(0.7)	-	-	-	-	-	-	−0.23*	−0.10
8. Morning negative emotions	1.5	(0.6)	-	-	-	-	-	-	-	−0.49*
9. Morning stress	1.2	(1.0)	-	-	-	-	-	-	-	-

N = 205 dreams, *p < 0.05.

### Main analyses

#### The discrepancy between subject and judge’s evaluations of positive and negative emotions in dreams

**Figure 1:**
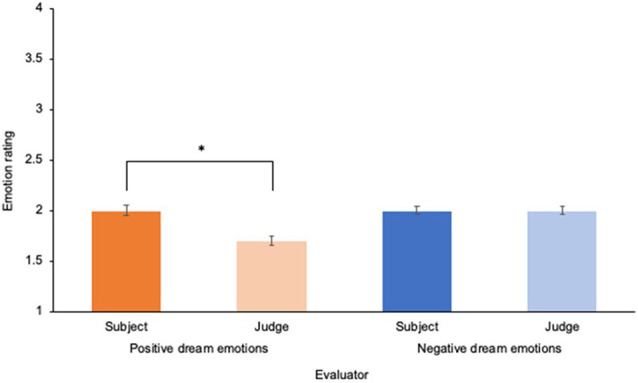
Mean differences in subject’s and a judge’s evaluations of positive and negative emotions in dreams. *N* = 345 dreams, ^*^p < 0.05. Error bars represent standard error of the mean.

The between-subjects MANOVA (*N* = 334) demonstrated that there were differences in the mean positive and mean negative emotional rating of the dream, *F*_(2,665)_ = 14.21, *p* < 0.001, $\eta_{p}^{2}$ = 0.041. This effect was driven by differences in subjects and judge’s mean scores in positive dream emotions only, *F*_(1,666)_ = 22.73, *p* < 0.001, $\eta_{p}^{2}$ = 0.033. As illustrated in Figure 1; Bonferroni corrected pairwise comparisons revealed that the subject’s positive dream emotion scores were higher than the judge’s positive dream emotion scores, *p* < 0.001. This suggests that subjects have a bias toward perceiving their dreams more positively than a judge, supporting the idea of a positivity bias. Furthermore, a Pearson’s correlation analysis demonstrated that the word length of the subject’s dream report was not significantly associated with their positivity bias score (*N* = 100; *r* = −0.16, *p* = 0.112).

#### Multi-group path analysis

**Figure 2:**
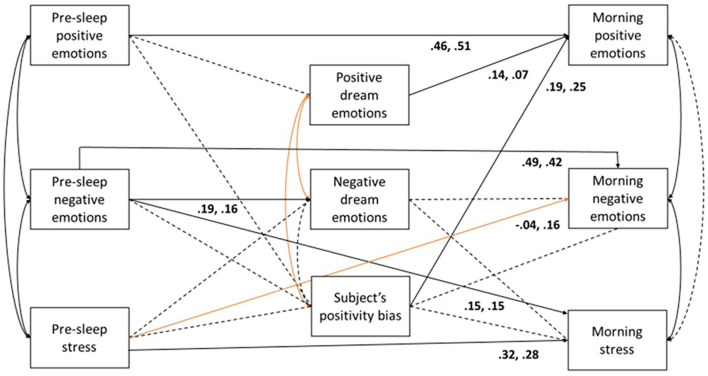
Multi-group path analysis model. Solid lines represent *p* < 0.05; dotted lines represent *p* > 0.05. Curved lines represent correlations; arrows represent regressed relationships. Orange lines represent paths that significantly differed between positive and negative dream nights. Coefficients are standardized and are presented in the following order: positive dream nights (*n* = 117 dreams) and negative dream nights (*n* = 205 dreams).

A model examining the associations between pre-sleep emotions and stress, dream emotions, and morning emotions and stress was examined (*N* = 322; *n* = 205 positive dreams, *n* = 117 negative dreams). The first model, in which the structural parameters were constrained to be equal across positive and negative dream nights, did not fit the data well (χ^2^_(46)_ = 94.22, *p* < 0.001, CFI = 0.87, TLI = 0.81, RMSEA = 0.08, SRMR = 0.09), rejecting the null hypothesis that the paths are equal across positive and negative dream nights. The χ^2^ diff test between the constrained and unconstrained model further suggested that some paths were different, *p* = 0.001. By unconstraining each path independently and conducting a χ^2^diff test, paths were different in the following relationships in those who had positive vs. negative dream nights: between pre-sleep stress and morning negative emotions (*p* = 0.020; pre-sleep stress was positively significantly associated with negative morning emotions in the negative dream nights only), between positive and negative dream emotions (*p* < 0.001; judge’s ratings of positive and negative dream emotions were negatively associated in positive dream nights only), and between positive dream emotions and subjects’ positivity bias (*p* < 0.001; judge’s ratings of positive dream emotions and subjects’ positivity bias was negatively associated in positive dream nights only). The final model with the aforementioned relationships unconstrained, allowing variability in the paths based on dream valence, is displayed in Figure 2. This model fit the data well and thus was retained, χ_(42)_^2^ = 56.89, *p* = 0.062, CFI = 0.96, TLI = 0.93, RMSEA = 0.05 (CI = 0.000–0.076), SRMR = 0.07. In those who had positive dreams, the following amount of variance was explained per construct: 0% in positive dream emotions, 5% in negative dream emotions, 0% in subject’s positivity bias, 26% in positive morning emotions, 23% in negative morning emotions, and 19% in morning stress. In those who had negative dreams, the following amount of variance was explained per construct: 0% in positive dream emotions, 3% in negative dream emotions, 0% in subject’s positivity bias, 34% in positive morning emotions, 30% in negative morning emotions, and 17% in morning stress.

As displayed in Figure 2, across both positive and negative dream nights, positive pre-sleep emotions were not associated with positive dream emotions or subjects’ dream positivity bias; however, positive pre-sleep emotions were significantly positively associated with positive morning emotions. Across negative and positive dream nights, pre-sleep negative emotions were significantly positively associated with negative dream emotions, morning stress, and negative morning emotions. There was no significant association between pre-sleep negative emotions and subjects’ dream positivity bias. Across negative and positive dream nights, pre-sleep stress was significantly positively associated with morning stress; however, pre-sleep stress was not associated with negative dream emotions or subjects’ dream positivity bias. In those who had negative dream nights, there was a positive association between pre-sleep stress and negative morning emotions, which was not present in those who had positive dream nights.

Regarding the associations between dream emotions and morning emotions, in those who had positive and negative dream nights, both positive dream emotions and subjects’ positivity bias were significantly positively associated with positive morning emotions. However, negative dream emotions and subjects’ positivity bias were not associated with negative morning emotions or morning stress. By examining the strength of the associations, irrespective of dream valence, the strongest predictor of positive morning emotions was pre-sleep positive emotions followed by subjects’ positivity bias. The strongest predictor of negative morning emotions was pre-sleep negative emotions. Finally, the strongest predictor of morning stress was pre-sleep stress followed by pre-sleep negative emotions.

## Discussion

This study sought to examine the relationships between bedtime mood and stress, dream mood, and waking mood and stress. We also sought to explore how an indicator of desensitization occurring in dreams (i.e., dreamer’s positivity bias) was associated with morning mood and stress. As observed in other research, dreamers perceived higher levels of positive emotions in their dreams compared to a judge even though most dreamers had more negative dream nights (Schredl and Doll, 1998; Sikka et al., 2014, 2018); however, there were no differences in the appraisal of negative emotions in the dream. Our results also suggest that mood and stress before sleep is the best predictor of mood and stress in the morning, respectively. However, the subject’s positivity bias is the second strongest predictor of positive morning emotions, suggestive of some evidence of mood regulation provided by desensitization during dreaming.

Congruent with our first prediction and with the observation of others (Schredl and Doll, 1998; Sikka et al., 2014, 2018), dreamers possess a bias that leads to the perception of a higher level of positive emotions in their dream compared to an objective evaluator (i.e., judge), which we refer to as a *positivity bias*. However, dreamer’s do not perceive a reduced level of negative emotions in their dream compared to a judge. This is consistent with previous works by others that demonstrate that subjects perceive a higher level of positive emotions in their dreams even when their dreams are objectively evaluated (by an independent judge) as negative (Schredl and Doll, 1998; Samson-Daoust et al., 2019). This finding is intriguing and suggests several phenomena at play. First, as seen in previous research, this difference could suggest that judges have more difficulty evaluating the positive emotions experienced by the dreamer in their dream narrative (Sikka et al., 2017; Lemyre et al., 2022). Interestingly, we did not find a correlation between the word length of the dream report and the degree of the subject’s positivity bias, potentially suggesting that the positivity bias is not created by methodological artifact but rather may represent an adaptive psychological response. For instance, it is plausible that this bias represents a mechanism that occurs during the dream experience as a result of the desensitization process. A similar adaptive response has also been found to be present in daytime mind wandering (Sikka et al., 2021).

In partial support of our third prediction, we observed that the discrepancy between the evaluation of positive emotions in the dream between the subject and the judge, termed as subjects’ positivity bias, played a positive role in morning emotions, supporting the emotion regulation function theories of dreams (Perlis and Nielsen, 1993; Malinowski et al., 2019). The positive relationship observed between the subject’s dream positivity bias and positive morning emotions may also be attributed to the feeling priming effect postulated by the Feeling Priming Theory (FPT; Lemyre et al., 2022) of dreams. According to FPT, feelings felt in dreams may be associated with feelings felt in waking due to the continued activation of dream feelings, in this case, heightened subjective perception of positive emotions, in memory during the postsleep period (Lemyre et al., 2022). In the multi-group path analysis model, we observed that subject’s positivity bias was the second strongest predictor of positive morning emotions; however, we did not observe any associations between the subject’s positivity bias and negative morning emotions or stress, which was incongruent with our predictions (Lemyre et al., 2022). Although the statistical significance of the path between the subject’s positivity bias and positive morning emotions was not significantly different between those who had more negative or positive dream nights, this association was still indeed stronger in those who had more negatively toned dreams, which partially supports our fourth prediction. This finding may suggest that emotion regulation processes are occurring in those who have more positive or negative dreams; however, this mechanism may be strengthened, perhaps through desensitization, as the dream increases in negative affectivity. As can be viewed in Tables s 1, 2, the bivariate correlations between subject’s positivity bias and morning mood are stronger in those who had more negative dreams: there was a larger negative correlation between subject’s positivity bias and negative morning emotions and a larger positive correlation between subject’s positivity bias and positive morning emotions. Furthermore, our null findings of the relative role of the subject’s positivity bias and morning stress may suggest that the mechanism underlying emotion regulation is distinct from the regulation of perceived stress. Further research is required to confirm this speculation. Taken together, these findings may be suggestive of a stronger mood regulation effect upon waking as a result of desensitization captured through the subject’s positivity bias when dreams increase in negative emotionality.

In relation to our predictions aligned with the CH, our findings partially support the notion of continuity between bedtime mood and stress, dream emotions, and waking mood and stress. Bedtime emotions and stress were the strongest predictors of waking emotions and stress irrespective of dream valence, suggestive of a certain degree of continuity between waking-life states after sleeping. We also observed that stress before sleep was positively associated with waking negative emotions but only in those who have negative dream nights. Furthermore, emotions and stress before sleep were not associated with dream emotions with the exception of negative bedtime emotions. This finding is relatively consistent with studies that suggest that salient emotions associated with everyday events are preferentially incorporated into dreams (De Koninck, 2012) and the presence of negative emotions at bedtime can be reflected in dreams (Malinowski and Horton, 2014). Furthermore, recent studies have shown that the best waking-life predictor of dream emotions is trait anxiety. Samson-Daoust et al. (2019) found that trait anxiety was the only factor that predicted the emotional tone of the dream through its interaction with other waking life factors, such as mood in the evening and stress during the day. This provides a nuanced understanding of the pattern of results we observed between pre-sleep and dream emotions such that there was a stronger degree of continuity between waking and dream affect when emotions were more negatively toned. Finally, dream emotions, with the exception of the dreamer’s positivity bias, were not associated with morning emotions or stress, which was partially incongruent with our predictions posited by the CH. It is plausible that these null findings were the result of our data analytical approach, such that we used the judge’s evaluations of the emotion levels in the dream as predictors, while also examining the variance explained by the subject’s positivity bias in morning mood and stress. From these results, it is clear that the subject’s evaluations of the emotions in their dream is a stronger predictor of their morning mood, especially when trying to predict positive morning emotions. Overall, our results offer some support for the CH, such that negative bedtime emotions can manifest into dreams, and that subject’s positivity bias, in addition to the subject’s bedtime mood and stress, is predictive of their waking mood and stress, respectively. The next step would be to link the positive bias to the actual content of dreams, most notably dream content characteristics identified in normative studies, such as aggression, failures, or misfortunes in addition to balance between positive and negative events in the dream (Dale et al., 2016) while using the same study design to examine whether the positivity bias is present and aids morning mood regulation through the desensitization to threats simulated in dreams.

### Limitations and future directions

Although the results of the present study provide some insight into theories of dream function, such as mood regulation, as well as insight into the relationships between waking-life and dream experience, they must be considered in light of certain limitations. First, the present study relied upon self-report measures for bedtime mood and stress, in the dream, and upon waking, and using a retrospective dream recall method. It is plausible that some subjects could have biases during their dream recall, which could impact the subjective evaluation of their emotions present in the dream and which dreams were reported. For instance, dream recall frequency and intensity of dream emotions are shown to enhance the feeling priming effect on waking states (Schredl, 2009). We consider this limitation to be somewhat unavoidable in dream studies, but it is nonetheless important to mention. Second, we did not use the same scales to evaluate emotions across all three time-points (bedtime, in the dream, and in the morning), which may have impacted the strength of the associations between them. For instance, we used the same scale for assessing emotions at bedtime and upon waking (i.e., emotion checklist) and a different scale to assess dream emotions (i.e., Hall and Van De Castle, 1966 rating system), which could have resulted in stronger associations between bedtime mood and morning mood and weaker associations between dream mood and morning mood. Due to this, we examined the strength and directionality of the correlations between the positive and negative emotions that were present in each scale and were measured across all three time-points (e.g., happiness, anxiety, anger, sadness, fear). We observed the same pattern of correlations present in Tables s 1, 2 when we examined the correlation of matched emotions over time (e.g., happiness at pre-sleep, evaluated in the dream, and at post-sleep), suggesting that our results are not due to a lack of systematic measurement of emotions across pre- and post-sleep and dreams. Furthermore, another factor that could have led to these patterns of results is the source of scoring for dream emotions. For instance, it is plausible that there would be stronger associations between subjects’ pre- and post-sleep emotions and dream emotions if we used subjects’ dream emotion scores in our model. However, due to the overlapping variance explained in positive morning emotions by subjects’ positive dream emotions score and their positivity bias score, this analytical technique was not pursued. Future studies should strive to replicate our findings using the same emotion checklists across time. Third, our sample consisted of individuals between the ages of 12 and 24, making our results difficult to generalize to the rest of the normative dream study sample. Future studies should strive to replicate our findings across all ages to understand the impacts of age on the degree of the subject’s positivity bias and its ability to contribute to mood regulation. Despite previous research suggesting that positive emotions reported by dreamers do not differ by developmental stages (Barbeau et al., 2022), it is still plausible that the subject’s may differ in their degree of positivity bias depending on their age. Finally, our results are correlational in nature; therefore, we cannot infer that the relationships that we observed are causal. Studies where the content of dreams is manipulated (De Koninck and Koulack, 1975; Koulack et al., 1985), would, when combined with the current methodology and data analytical technique, allow us to directly assess the adaptive role of the subject’s positivity bias on waking mood regulation. More importantly, from a theoretical perspective, our results reflect the impact of remembered dreams while most dreams in day-to-day life are not recalled. Most theories, including the most recent formulations (Lemyre et al., 2022; Wamsley, 2022) do not address this important matter; however, it is interesting to note that Freud postulated that dreams were the “Guardians of Sleep” and that dreams that are remembered have failed their function. A similar point is made by Kramer (2007) who also suggested that mood regulation is best achieved during undisrupted sleep with the normal REM sleep components. Despite this notion, the desensitizing mechanism that is thought to be attributed to muscle relaxation during REM (Perlis and Nielsen, 1993) or its neuronal basis (Scarpelli et al., 2019; Sikka et al., 2021) is thought to still apply to non-remembered REM dreams (De Koninck, 2012). It is thus possible that the observed desensitization observed here in dream mood reflects the more important desensitization attributable to REM sleep itself. Future research is required to understand potential mechanistic differences, including differential impacts on affect regulation, in the desensitization process in remembered and non-remembered dreams.

### Conclusion and practical implications

Our results lend partial support to emotion regulation theories of dream function and to the CH. We observed that subjects possess a positivity bias as indicated by a discrepancy between dreamers and a judge’s evaluations of positive emotions in the dream. In turn, this bias was positively associated with positive morning emotions; stronger associations between these constructs were found in those who had more negatively toned dreams. These findings support the adaptive role of the subject’s positivity bias in recalled dreams on their waking emotions. They also shed light on the potential mechanism by which desensitization, as indicated by this bias, supports emotion regulation. These findings are particularly relevant in understanding the importance and function of the dream experience in dampening the emotional tone of traumatic memories in individuals who may have compromised emotion regulation and fear extinction mechanisms during wakefulness due to their exposure to an adverse event in waking-life (Scarpelli et al., 2019). Future research should examine whether these individuals demonstrate a dream positivity bias and its respective implications on their subsequent waking mood. Furthermore, our results may also have neurodevelopmental implications given that the participants in our sample were adolescents and young adults. Previous work demonstrates that adolescents and young adults have more nightmares compared to older adults (Salvio et al., 1992; Schredl and Doll, 1998; Nielsen et al., 2006); however, we observed that despite our sample having more negatively toned dreams, many demonstrated a dream positivity bias. It is plausible that the presence of this bias or its magnitude is attributed to a compensatory mechanism that aids psychological adaptation during developmental periods where fear conditioning and extinction are still developing, such as in adolescence (Shechner et al., 2014). Therefore, the positivity bias may be indicative of an adaptive associative learning mechanism that is reflected in the dream experience, which pairs higher levels of positivity affectivity with simulations of feared waking events to help downregulate neuronal fear responses to these events during wakefulness during a developmental period characterized by overactive fear responding and weak functional connectivity between brain regions involved in the fear response (i.e., amygdala) and emotion regulation (i.e., pre-frontal regions; Somerville et al., 2010).

## Data Availability Statement

The raw data supporting the conclusions of this article will be made available by the authors, without undue reservation.

## Ethics Statement

The studies involving human participants were reviewed and approved by Research Ethics Board of the University of Ottawa. Written informed consent to participate in this study was provided by the participants’ legal guardian/next of kin.

## Author Contributions

JD, KB, CT, and AL: all contributed to the conception and design of the study. CT, AL, and EC: contributed to the data gathering and dream scoring. CT: collated the data in preparation of the statistical analyses, wrote an Honours Thesis in French based on part of this study. KB and CT: carried out the statistical analyses of the dreams, carried out the statistical analyses. KB, CT, JD, and AL: prepared the final manuscript. JD: obtained the funding. All authors contributed to the article and approved the submitted version.

## Funding

This work was supported by a grant from the Social Sciences and Humanities Council of Canada (435-2020-0851).

## Conflict of Interest

The authors declare that the research was conducted in the absence of any commercial or financial relationships that could be construed as a potential conflict of interest.

## Publisher’s Note

All claims expressed in this article are solely those of the authors and do not necessarily represent those of their affiliated organizations, or those of the publisher, the editors and the reviewers. Any product that may be evaluated in this article, or claim that may be made by its manufacturer, is not guaranteed or endorsed by the publisher.
